# Fast-growing pancreatic neuroendocrine carcinoma in a patient with multiple endocrine neoplasia type 1: a case report

**DOI:** 10.1186/1752-1947-2-354

**Published:** 2008-11-18

**Authors:** Jens Waldmann, Nils Habbe, Volker Fendrich, Emily P Slater, Peter H Kann, Matthias Rothmund, Peter Langer

**Affiliations:** 1 Department of General Surgery, University Hospital Giessen and Marburg, Marburg, Baldingerstrasse, 35037 Marburg, Germany; 2 Department of Internal Medicine, Division of Endocrinology and Diabetology, University Hospital Giessen and Marburg, Baldingerstrasse, 35037 Marburg, Germany

## Abstract

**Introduction:**

Predictive genetic screening and regular screening programs in patients with multiple endocrine neoplasia type 1 are intended to detect and treat malignant tumors at the earliest stage possible. Malignant neuroendocrine pancreatic tumors are the most frequent cause of death in these patients. However, the extent and intervals of screening in patients with multiple endocrine neoplasia type 1 are controversial as neuroendocrine tumors are usually slow growing. Here we report the case of a patient who developed a fast-growing neuroendocrine carcinoma within 15 months of a laparoscopic distal pancreatic resection.

**Case presentation:**

We followed a group of 45 patients with multiple endocrine neoplasia type 1 by an annual screening program in the Department of Visceral, Thoracic, and Vascular Surgery at the University Hospital Marburg in cooperation with the Department of Radiology and the Division of Endocrinology. A man with multiple endocrine neoplasia type 1 who was diagnosed with a recurrent primary hyperparathyroidism underwent a distal pancreatic resection for a non-functional neuroendocrine tumor. In the context of our regular screening program, a large non-functional neuroendocrine tumor was diagnosed in the pancreatic head 15 months after the first pancreatic surgery. Therefore, we performed an enucleation and regional lymph node resection. At histology, the diagnosis of a neuroendocrine carcinoma with one lymph node metastasis was established. There was no evidence of recurrence 9 months after re-operation.

**Conclusion:**

Fast-growing neuroendocrine tumors are rare in patients with multiple endocrine neoplasia type 1. The intervals, both postoperative and in newly diagnosed pancreatic lesions, in patients with multiple endocrine neoplasia type 1 should be reduced to 6 months to establish the early diagnosis of rapidly progressive disease in a small subset of patients.

## Introduction

Multiple endocrine neoplasia type 1 syndrome (MEN1) is an inherited tumor syndrome, which is typically characterized by tumors of the parathyroid glands, the pancreas and the pituitary. Organs such as the adrenal glands, the thymus, the skin and the bronchial tree are involved less frequently [1-4]. Pancreatoduodenal endocrine tumors (PETs) are determinants of long-term survival. About one-third of patients with MEN1 develop malignant tumors [5,6]. Predictive genetic screening and regular screening programs are designed to detect and treat malignant tumors at the earliest stage possible. However, the extent and intervals of screening in patients with MEN1 are controversial. Current recommendations are based on the National Institutes of Health (NIH) consensus conference in 2001 [7]. A yearly biochemical screening and a tumor imaging every 3 to 5 years are emphasized. In the past few years, endoscopic ultrasound has gained importance in the detection of PETs [8,9], particularly in the setting of a prospective screening program. To date, the survival benefit of periodic screening and early intervention has not been proven.

As a consequence of periodic screening, asymptomatic patients with functioning or non-functioning tumors are scheduled for pancreatic resections more often and at a younger age. The indication, extent and timing of surgery in patients with MEN1 are a matter of debate. Indications for insulinomas and non-functioning tumors larger than 2 cm are well established, although some groups postulate a more aggressive strategy with a limit of 10 mm in non-functioning pancreatoduodenal endocrine tumors (nf PETs) in order to prevent malignancy and liver metastases [10-12]. Malignancy is evident in about one-half of patients with PETs; unfortunately, markers for the development or progression of malignant disease are not yet available.

Here we report the case of a 37-year-old man with MEN1 who developed a rapidly growing non-functioning tumor in the pancreatic remnant 15 months after a laparoscopic distal pancreatic resection. To the best of the authors' knowledge, this is the first report of a rapidly growing non-functioning PET in a patient with MEN1 that was detected by a regular screening program.

## Case presentation

**Figure 1:**
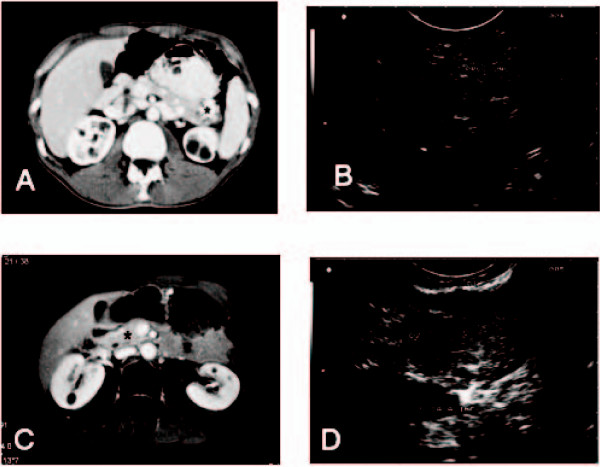
**Imaging studies before initial and re-operation**. (A) Pre-operative computed tomography scan before the initial operation. (B) Pre-operative endoscopic ultrasound before the initial operation (asterisk indicates the tumor; Proc. unc., normal appearing uncinate process). (C) Pre-operative computed tomography scan before the re-operation. (D) Pre-operative endoscopic ultrasound before the re-operation.

A 37-year-old man was diagnosed with MEN1 owing to a recurrent symptomatic primary hyperparathyroidism (pHPT) and a positive family history. Genetic analysis of the *Menin *gene showed a frame-shift mutation in codon 229 (c.657 1bpdel/K285X). At the initial evaluation, a non-functioning pituitary gland tumor, non-functioning adrenal lesions and a 17 mm non-functioning PET were detected. Normal levels of pancreatic polypeptide, gastrin, chromogranin A, serotonin, insulin, proinsulin and glucagon were present. Computed tomography (CT) showed a tumor in the pancreatic tail with a diameter of 25 mm without any radiological signs of malignancy. Endoscopic ultrasound (EUS) visualized two small lesions in the pancreatic tail (Figure 1A). Somatostatin-receptor scintigraphy (SRS) did not provide any evidence of a somatostatin-receptor positive tumor. There was no evidence of lymph node metastases (LNMs) or distant metastases (DMs) in the imaging procedures.

This patient was scheduled for a laparoscopic distal pancreatic resection after laparoscopic ultrasound (LUS) to rule out additional tumors in the pancreatic head and body. At laparoscopy, after mobilization of the pancreatic tail, LUS confirmed the finding of the pre-operative EUS. In the absence of additional lesions in the pancreatic head and body, as well as no DMs or LNMs, the patient underwent a spleen-preserving distal pancreatic resection. A rapid histological diagnosis was performed to exclude malignancy. Seven days after an uneventful clinical course, the patient was discharged.

**Figure 2:**
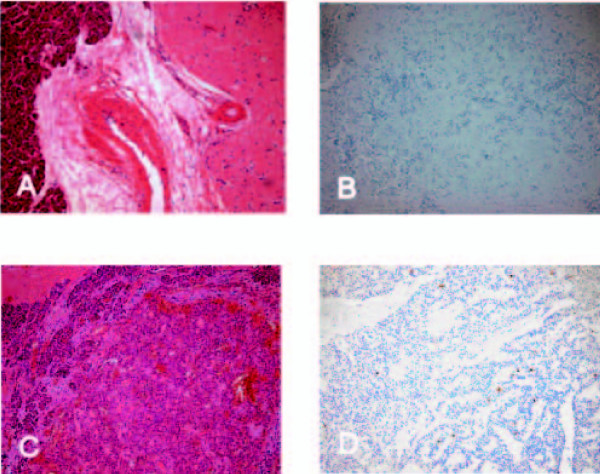
**Histological characteristics of the neuroendocrine tumor at initial operation and the neuroendocrine carcinoma of the pancreas at re-operation**. (A) Hematoxylin and eosin stain of the large neuroendocrine tumor at the initial operation. (B) Ki-67 stain of the large neuroendocrine tumor at the initial operation. (C) Hematoxylin and eosin stain of the neuroendocrine carcinoma at re-operation. (D) Ki-67 stain of the neuroendocrine carcinoma at re-operation.

Histopathological examination showed four well-differentiated neuroendocrine tumors (one of 40 mm, three of 3 mm). Immunohistochemistry displayed a positive staining for synaptophysin and chromogranin A. The Ki-67 index was lower than 1% (Figure 2A).

The patient was re-evaluated 15 months after surgery. At presentation, the patient was asymptomatic. Thus, he participated in our regular screening program and magnetic resonance imaging (MRI), SRS and EUS were performed. As was the case before the initial operation, hormone levels were within the normal range. Surprisingly, MRI and EUS demonstrated a tumor measuring 25 mm in the pancreatic head and an enlarged lymph node (LN) above the caval vein on the lower pancreatic margin. DMs were not detected. An additional pancreatic lesion, 7 mm in diameter, was visualized by EUS (Figure 1B). The pancreatic head tumor had developed within 12 months of surgery, but radiological signs of invasion could not be established. The patient was scheduled for surgical exploration as malignancy was suspected owing to the rapid growth.

**Figure 3:**
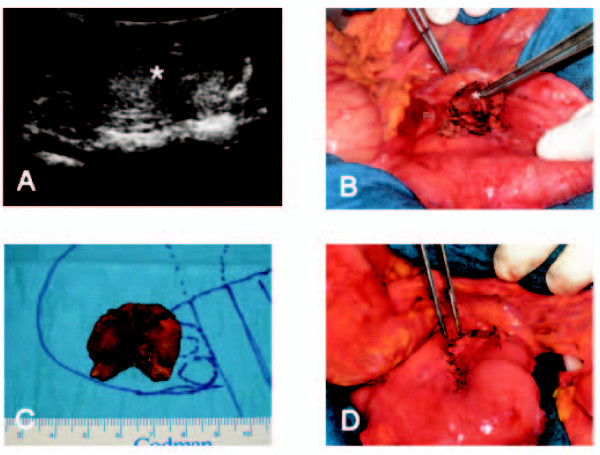
**Situs at laparotomy before and after resection**. (A) Intra-operative ultrasound at laparotomy. (B) Situs at laparotomy (asterisk indicates the tumor; S, stomach; P, pylorus; PH, pancreatic head; D, duodenum). (C)Macroscopic view of the resected tumor. (D) Covering of the defect after resection by a Y-en-Roux jejunal loop.

At laparotomy, an intra-operative ultrasound (IOUS) was performed (Figure 3A). A well-outlined encapsulated tumor measuring 22 mm was found in the pancreatic head. Before the final decision on the surgical procedure was made, one enlarged, but soft, LN of 15 mm from the hepatic ligament was transected to rule out LNMs. As rapid histological diagnosis showed a normal LN, the pancreatic head tumor was enucleated. At the lower margin of the pancreatic head, two enlarged LNs of 10 mm were also transected. The tumor bore macroscopic invasion of the surrounding pancreatic tissue. The defect was covered by a Y-en-Roux loop (Figure 3B). A resection of the pancreatic head would have resulted in a pancreatectomy, which results in XXX pancreoprivic diabetes. The patient was discharged after an uneventful clinical course of 10 days.

Gross examination showed a pale tumor 28 mm in diameter and a smooth, lobulated cut surface. The tumor was covered by a white capsule (Figure 3C). Microscopically, the tumor-forming epithelial glands infiltrated the pancreatic tissue. The tumor cells showed a hyperchromatic nucleus with a gross chromatin structure. In some parts, the tumor displayed necrosis. Immunohistochemistry resulted in a positive staining for chromogranin A and a negative staining for insulin and gastrin. The Ki-67 index was again lower than 1%. The LN of the hepatic ligament (12 mm) and one of the two LNs of the lower pancreatic margin (10 mm) were without evidence of tumor cells. The second LN of the lower pancreatic margin, measuring 9 mm, revealed an infiltration of atypical epithelial cells.

## Discussion

In our experience with more than 40 patients who participate in a regular screening program at our hospital, this is an extraordinary case. Most non-functioning PETs in patients with MEN1 are small, multiple and follow a benign course and, consequently, are seen as slow-growing tumors [13].

It is noteworthy that malignant PETs have become the most important determinant of long-term survival [5,6]. About one-third of patients with MEN1 succumb to malignant tumors. To date, no markers for malignancy have been established. As a consequence, the rationale for screening is to detect lesions at an earlier stage and to perform prophylactic, but pancreas-preserving, surgery before DMs or LNMs develop. Predictive genetic screening and regular screening programs are intended to detect and treat malignant tumors at the earliest stage possible. However, the extent and intervals of screening in patients with MEN1 are controversial owing to the fact that a survival benefit of periodic screening and early intervention has not been proven. Most authors emphasize a postoperative follow-up after 12 months [14]. The Uppsala group and the NIH consensus conference suggest a regular screening interval of 3 to 5 years [7,15].

In view of the presented case, the diagnosis of a new, rapidly growing neuroendocrine carcinoma was established in an asymptomatic patient as a result of a postoperative follow-up at 15 months. A misdiagnosis and oversight by three different imaging modalities (CT, EUS and IOUS) before the initial surgery seem to be unlikely. A 6-month follow-up in our patient would possibly have resulted in earlier surgery on a smaller tumor. Therefore, we suggest a closer follow-up every 6 to 12 months, postoperatively in the case of a newly diagnosed lesion, taking into consideration that a small subset of patients seems to develop rapidly progressive disease. However, this policy leads to a higher number of 'unnecessary investigations' in patients who follow a benign course. Once patients display stable disease over several years, the intervals may be extended. Prospective data on follow-up of PETs in patients with MEN1 are rare, and most studies did not differentiate between prospective and non-prospective diagnosed PETs. However, randomized prospective studies seem to be unethical owing to the potential benefit of regular screening.

The indication for surgery in patients with MEN1 is an unresolved controversy. The observation that non-functioning PETs smaller than 3 cm rarely developed LNMs and DMs has prompted some groups to suggest operating only on PETs larger than 3 cm. However, one could argue that the aim is not to detect but to prevent metastases, which leads to a more aggressive strategy and indicates surgery when a PET larger than 10 mm is detected. Skogseid and Oberg emphasize performing surgery when biochemical evidence is established with or without positive imaging results [10].

The extent of surgery in non-functioning PETs is controversial, although distal pancreatic resection up to the level of the portal vein and enucleation of pancreatic head tumors including LN transection is the procedure most groups prefer. Laparoscopic resection could also be considered, simply because there are no data on the value of routine LN dissection, notably in the setting of early surgery in small non-functioning PETs. Whenever a re-operation is indicated, the strategy must be tailored to the patient, which makes it difficult to establish 'guidelines': the younger the patient is, the more pancreatic tissue must be preserved to prevent XXXpancreoprivic diabetes. In elderly patient or in patients who present with diabetes, even pancreatectomy is sometimes indicated, for example, after a distal pancreatic resection when the tumor is located in the pancreatic head. The location, number and size of tumors must be taken into consideration to 'design' tailored surgery in patients with MEN1.

Whenever dealing with prophylactic surgery, low morbidity and almost absent mortality is required. It has to be emphasized that most patients with non-functioning PETs are asymptomatic and have excellent long-term survival without surgery. Therefore, total pancreatectomy is rarely indicated but has been advocated for patients in families with aggressive PETs. In our experience, it must be considered that most patients who have undergone pancreatic resection are discovered in the follow-up to have developed small non-functioning PETs, which would lead to additional surgery in the future. Patients with a prior distal pancreatectomy would consequently undergo a total pancreatectomy. The XXXpancreoprivic severe diabetes in a patient of 40 or 50 years is of utmost importance.

The patient we have presented here was scheduled for an exploration, which could have resulted in either an enucleation or a pancreatoduodenectomy. A resection of the pancreatic head preserving the duodenum was also an option. After the rapid section of the LN in the hepatic ligament excluded LNMs, we decided on an enucleation of the soft PET in the pancreatic head. Surprisingly, one LN, macroscopically unsuspicious, at the lower margin was an LNM. We will follow the patient closely and if he displays evidence for local recurrence, he will be scheduled for total pancreatectomy. Six months after surgery, he was without any evidence of recurrence.

## Conclusion

The postoperative follow-up intervals and those for newly diagnosed pancreatic lesions should be reduced to 6 months to establish diagnosis as soon as possible in patients with rapidly progressing disease.

## Abbreviations

CT: computed tomography; DM: distant metastasis; EUS: endoscopic ultrasound; IOUS: intra-operative ultrasound; LN: lymph node; LNM: lymph node metastasis; LUS: laparoscopic ultrasound; MEN1: multiple endocrine neoplasia type 1 syndrome; MRI: magnetic resonance imaging; PET: pancreatoduodenal endocrine tumor; NIH: National Institutes of Health; PET: pancreatoduodenal endocrine tumor; SRS: somatostatin-receptor scintigraphy

## Consent

Written informed consent was obtained from the patient for publication of this case report and any accompanying images. A copy of the written consent is available for review by the Editor-in-Chief of this journal.

## Competing interests

The authors declare that they have no competing interests.

## Authors' contributions

JW selected the case and drafted the manuscript. NH drafted the manuscript and critically revised the manuscript for important intellectual content. VF participated in the discussion and critically revised the manuscript for important intellectual content. EPS, PHK, and MR critically revised the manuscript for important intellectual content. PL selected the case and critically revised the manuscript for important intellectual content.
